# Major aging-associated RNA expressions change at two distinct age-positions

**DOI:** 10.1186/1471-2164-15-132

**Published:** 2014-02-14

**Authors:** Marius Gheorghe, Marc Snoeck, Michael Emmerich, Thomas Bäck, Jelle J Goeman, Vered Raz

**Affiliations:** 1 Department of Human and Clinical Genetics, Leiden University Medical Centre, Leiden, The Netherlands; 2 Department of Medical Statistics, Leiden University Medical Centre, Leiden, The Netherlands; 3 Leiden Institute of Advanced Computer Science, Leiden University, Leiden, The Netherlands; 4 Department of Anaestasia, Canisius-Wilhelmina Hospital, Nijmegen, The Netherlands; 5 Biostatistics, Department for Health Evidence, Radboud University Medical Center, Nijmegen, The Netherlands

**Keywords:** Human aging, Expression profiles, Quadratic regression model, Kmeans clustering

## Abstract

**Background:**

Genome-wide expression profiles are altered during biological aging and can describe molecular regulation of tissue degeneration. Age-regulated mRNA expression trends from cross-sectional studies could describe how aging progresses. We developed a novel statistical methodology to identify age-regulated expression trends in cross-sectional datasets.

**Results:**

We studied six cross-sectional RNA expression profiles from different human tissues. Our methodology, capable of overcoming technical and genetic background differences, identified an age-regulation in four of the tissues. For the identification of expression trends, five regression models were compared and the quadratic model was found as the most suitable for this study. After *k-means* clustering of the age-associated probes, expression trends were found to change at two major age-positions in brain cortex and in *Vastus lateralis* muscles. The first age-position was found to occur during the fifth decade and a later one during the eighth decade. In kidney cortex, however, only one age-position was identified correlating with a late age-position. Functional mapping of genes at each age-position suggests that calcium homeostasis and lipid metabolisms are initially affected and subsequently, in elderly mitochondria, apoptosis and hormonal signaling pathways are affected.

**Conclusions:**

Our results suggest that age-associated temporal changes in human tissues progress at distinct age-positions, which differ between tissues and in their molecular composition.

## Background

Biological aging represents an age-dependent decline of multiple physiological functions, tissue degenerations and molecular changes. In higher organisms, the aging process is determined by a combination of genetics and environmental factors, and therefore is considered as a complex process. The complexity of the biological aging is also contributed to by multiple molecular inputs [1]. Molecular regulation and regulators of aging have been identified in simple animal models. However, it is still unclear whether they have a similar contribution to human aging [2]. In contrast to simple animals, the aging of humans is characterized by high variations. In order to understand this process, robust and unbiased statistical analyses should be developed.

To describe aging as a time-dependent process, changes in physiological, cellular or molecular parameters with chronological age should be elucidated. For an accurate description, these parameters must be quantitative and should be analyzed with appropriate statistical tests. Ideally, the rate of aging-associated changes would be extracted from longitudinal datasets, where changes in one subject are followed over the course of time. In humans, however, longitudinal sampling is often impractical. Instead, a cross-sectional dataset that covers a broad age-range is an alternative [2].

Cross-sectional genome-wide RNA datasets were generated from healthy subjects. Genes contributing to tissue degeneration have been identified (reviewed in: [2,3]). In these studies, linear models or an age group analyses were applied to identify age-regulated genes [4-8]. Linear models are simple and easy to apply, but make many assumptions that are doubtful for complex processes, such as aging. When describing a time-dependent process with a linear model, we assume a constant change over the entire time, which is highly unexpected in biological processes. To investigate when aging initiates and how it progresses, additional models should be considered.

**Figure 1:**
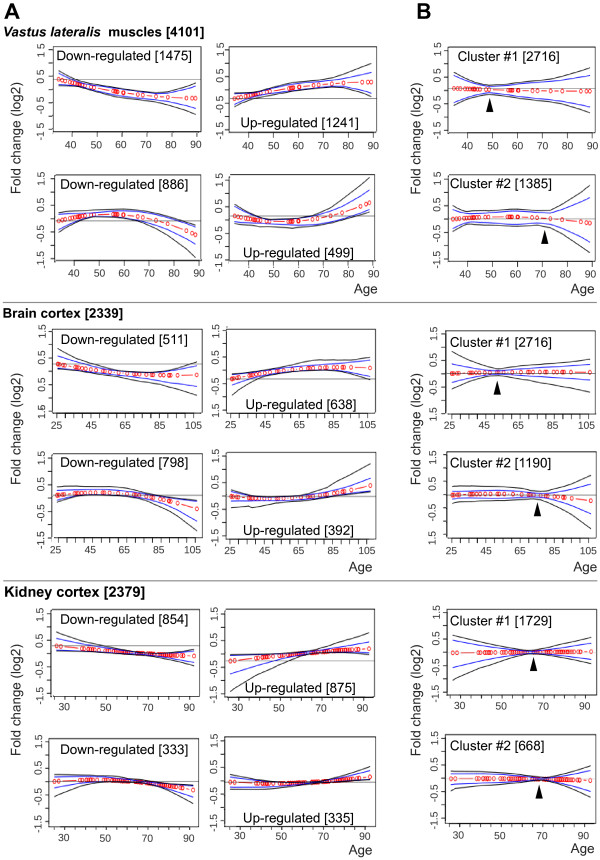
**Dominant expression trends in *VL muscles*, brain cortex and kidney cortex.** Plots show the major trends identified from the significant age-regulated probes using *k-*means with Euclidean distance as metric **(A)** or absolute correlation as metric **(B)**. The X-axis represents the age in years and the Y-axis the fold change (log2) after normalization to the average. Shown are the major age-regulated trends, where red circles interconnected by red lines represent the cluster centroids, blue and black lines are the 95th or 99th percentiles, respectively. The number of probes in each cluster is indicated between brackets. Arrowheads indicate the age-positions.

We developed a novel five-step methodology to study temporal changes in cross-sectional genome-wide RNA datasets. In this methodology, the expression trend of each probe was determined using a quadratic model and its *p*-value was calculated in order to filter the age-regulated probes. Subsequently, the age-regulated probes were clustered such that major age-dependent expression trends could be identified (outlined in Figure 1). The methodology was developed using a new *Vastus lateralis* (*VL*) muscles microarray dataset and was evaluated and validated on post-mortem brain frontal cortex (brain cortex) [8], kidney cortex and medulla [6] (Table 1). Despite technical and genetic background differences between platforms, similar age-regulated trends were found in *VL* muscles and in brain cortex. In both datasets, the expression trends bend at two distinct age-positions, at midlife and in old age. In kidney cortex, however, trends bend only in old age. Based on these results we suggest that during aging, significant changes in RNA expression progress through distinct time points, which differ molecularly.

**Table 1:** Description of the datasets used in this study and the statistical evaluation of age-associated probes

**Tissue**	**Chronological datasets**				**Permuted datasets (N=100)**	
	**Nr. samples**	**Age range**	GT p-value *	Age-regulated **	Age-regulated mean ± SE**	**[Ps>Cs/Ps<Cs]^**
*VL muscles*	29	35-89	0.015	4101	2991 ± 86.1	7/93 [0.07]
Brain cortex	30	26-106	0.004	2339	677 ± 52.2	3/97 [0.03]
Kidney cortex	72	27-92	0.002	2397	1136 ± 77.2	11/89 [0.12]
Kidney medulla	61	29-92	0.205	1474	1090 ± 70.0	24/76 [0.31]

Table shows the four datasets used in this study in chronological order and permuted samples. The number of samples and the age range (in years) are indicated.

* The *p*-value determined with the *globaltest* (GT).

** The number of age-regulated probes (*p*-value < 0.05) was determined using a quadratic regression model and no age-association as null hypothesis. In the permuted datasets, the mean and the standard error (SE) from 100 permutations per dataset were calculated.

^The ratio between permuted samples (Ps) with a higher to lower number of significant probes, compared with chronologically ordered samples (Cs).

## Results

### Identification of age-regulated datasets and age-regulated genes

Several cross-sectional microarray studies in humans are publically available [3]. Those were generated on different platforms and present different genetic backgrounds and technical variations in RNA isolation and labeling. We analyzed RNA microarray datasets from *VL* muscles of healthy individuals [9] brain cortex [8], kidney cortex and medulla [6] (datasets are detailed in Additional file 1: Table S1). The datasets were separately analyzed using a similar procedure: normalization using Variance Stabilization and Normalization (VSN), log transformation and gender correction. A significant age-association (*p*-value < 0.05) was found in *VL* muscles, brain and kidney cortex but not in kidney medulla (Table 1). Importantly, genetic background differences and technical variations are not expected in the kidney cortex and medulla datasets, since both are from the same platform and in most cases, tissues were collected from the same individuals [6]. Despite genetic background and technical differences between *VL* muscles and brain cortex, in both datasets the age-association of genome-wide expression profiles is the most robust. This analysis indicates that reliable age-regulated probes would be found in *VL* muscles, brain and kidney cortex, but not in kidney medulla.

We have used the datasets from *VL* muscles, brain cortex and kidney cortex to develop a methodology for age-associated RNA expression trends identification, while the kidney medulla dataset was used as a methodological control. This analysis indicates that in humans age-associated genome-wide expression profiles highly differ between tissues.

### Identification of expression trends in cross-sectional datasets

In cross-sectional datasets, expression levels can vary between individuals and could constrain the identification of significant age-associated expression trends. To overcome this limitation, expression levels were smoothed per probe prior to the selection of age-associated probes. The probe smoothing was carried out per dataset. We compared five different smoothing procedures: linear, quadratic and cubic regression models in order to identify the most adequate for cross-sectional gene expression datasets (Additional file 1: Figure S1A). The genome-wide fitness of each regression model was statistically evaluated for all probes, per platform and the fitness was compared between each two models. This analysis indicates that the cubic and quadratic model have a similar fit, whereas the fitness of the linear model was significantly worse (Additional file 1: Figure S1B). The robustness of each model was assessed using the ‘leave-one-out’ cross-validation, indicating that between the four regression models, the linear model has the largest error rate (Additional file 1: Figure S1B). Together, these analyses indicate that a linear model is less suitable to describe age-associated changes in cross-sectional datasets, compared with quadratic or cubic models. It is important to note that quadratic and cubic regression models use fewer assumptions than the linear model, therefore would be more suitable for a dataset in which complex changes occur with time. Although both quadratic and cubic models showed similar fit, we carried our analysis using the quadratic regression model, as it is less likely to overfit the data, in comparison with the cubic model.

The significance of age-regulated trends was determined per probe, assuming no age-association as the null hypothesis. Only probes presenting a *p-*value < 0.05 (unadjusted) were kept for further analysis (Table 1 and Additional file 1: Figure S2). Each dataset passed two filtering steps, therefore *p*-values were not corrected for multiple testing. In accordance with the *globaltest p*-value, the number of age-regulated probes in brain, *VL* muscles and kidney cortex was higher than in kidney medulla, which did not pass the *globaltest p*-value threshold (Table 1). Only limited overlap (~10%) of age-regulated genes was found between each two-tissue combination and less than 2% overlap was found between three-tissue combinations (Additional file 1: Table S3). This suggests that molecular aging differs among tissues, therefore the analysis was carried out per tissue.

To statistically assess the smoothing procedure and the significance of the age-regulated probes, the samples in each dataset were randomly permuted, generating artificial datasets. One hundred permuted datasets were generated per tissue, in order to reduce the impact of permutations that do not highly differ from the chronologically ordered dataset. The ratio between higher to lower numbers of significant probes in permuted datasets compared with the chronologically ordered dataset was considerably small in brain, *VL muscles* and kidney cortex (Table 1). In contrast, in the kidney medulla the ratio was much higher (0.31, Table 1), suggesting that the age-regulated probes in this dataset may represent noise.

The number of probes that are found in the original data, but not in the average permuted dataset is an estimation of the amount of truly aging-associated genes [10], therefore from the dataset permutation analysis we can conclude that in the significantly age-regulated datasets the ratio signal to noise is very small.

This analysis demonstrates that the filtering step of the age-regulated probes from the chronologically ordered datasets can be performed with a significant confidence, even without correction for multiple testing. In addition, this suggests that in the datasets with no significant age-association, the fraction of false-positive age-associated probes would be high. Moreover, our procedure for age-associated probe identification is not highly affected by genetic background and platform differences.

### Identification of major age-associated expression trends and bend-positions

To identify trends that are significantly associated with chronological age, the filtered probes were clustered using *k*-means clustering, with Euclidean distance as metric. We identified four stable clusters exhibiting unique trends (Figure 1A and Additional file 1: Figure S3B). Interestingly, reciprocal trends bend at similar age (Figure 1A), suggesting that major expression changes are at defined time points. To extrapolate the age at which major expression changes occur, we applied *k-*means clustering with absolute correlation as metric, which clusters reciprocal trends together. Two major trends were identified (Figure 1B). Each absolute correlation cluster represents two reciprocal trends and the time point at which the reciprocal trends intersect within the cluster, represents the age where trends bend (Figure 1B). In addition, this point is also defined as the smallest distance between the 95th percentile and the cluster centroids (Figure 1B). We named this point an age-position. In *VL* muscles, two age-positions were identified: at age 43 ± 3 and 75 ± 5 (Table 2A). The range of each age-position was estimated from 2, 3 and 4 *k*-means absolute correlation clusters. Moreover, in brain cortex two distinct age-positions at 53 ± 3 and 77 ± 3 years were identified (Table 2A and Figure 1B). In contrast, in kidney cortex the two age-positions were indistinguishable by age (Table 2A). This analysis suggests that age-positions spatially and temporally differ between tissues.

**Table 2:** Probes associated with an age-position and the gene overlap in age-positions

**A**	**Dataset details**		**1st age-position**		**2nd age-position**	
**Tissue**	**Nr samples**	**Age-range**	**Age**	**# Probes**	**Age**	**# Probes**
				**up | down**		**up | down**
***VL muscles***	29	35-89	43±3	2716	75±5	1385
				1241 | 1475		499 | 886
**Brain cortex**	30	26-106	53±3	1149	77±3	1190
				638 | 511		392 | 798
**Kidney cortex**	72	27-92	65±5	1729	70±5	668
				392 | 854		335 | 333
**B**	**1st age-position**			**2nd age-position**		
	*VL* muscles		Brain cortex	*VL* muscles		Brain cortex
***VL *** **muscles**	1976 (100%)		95 (9.4%)	1094 (100%)		86 (6.4%)
**Brain cortex**	95 (4.8%)		1010 (100%)	68 (6.2%)		1057 (100%)

**A.** Table summarizes the age (in years) of each age-position and the number of probes, as well as the direction of regulation (up or down) in *VL* muscles, brain cortex and kidney cortex. Age-positions ± variations in years are indicated.

**B.** Table shows the gene overlap between *VL* muscles and brain cortex in each age-position. Gene overlap was determined with Entrez ID. The percentage of overlap is indicated between brackets.

**Figure 2:**
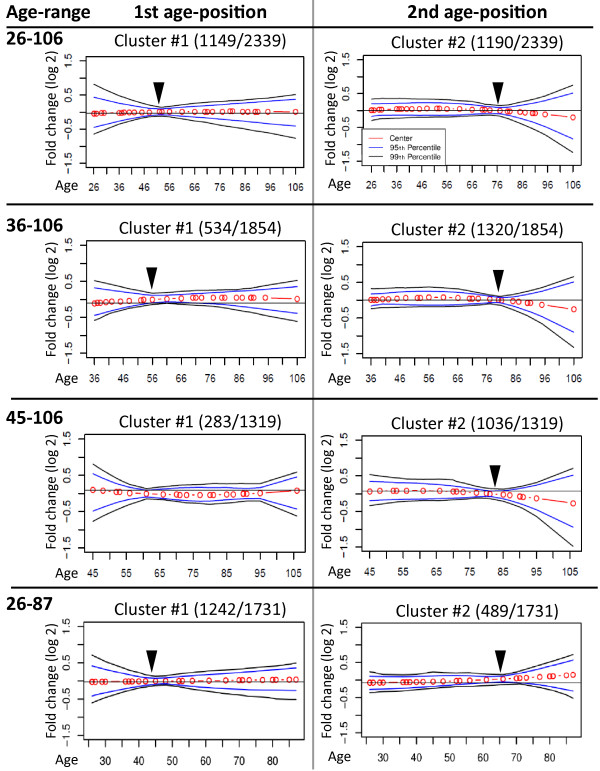
**The effect of the dataset age-range on age-positions.** Plots show the expression trends of the significant probes clustered using *k-*means with absolute correlation as metric in the brain cortex dataset. Trends were generated for the age-ranges: 26–106 (original); 36–106; 45–106 and 26–87. Red circles interconnected by red lines represent the cluster centroids, blue and black lines are the 95th or 99th percentiles, respectively. The number of probes in each cluster is indicated between brackets. Arrowheads denote the age-positions, indicating a bend in the expression trend.

To assess this aspect, we generated from the brain cortex dataset (26–106 years) three datasets with varied age ranges: 36–106, 45–106 and 26–87. The analysis was repeated on these variant datasets starting from the probe smoothing step. The occurrence of the first age-position in the datasets ‘trimmed’ from the left (youngest samples) was shifted around three years to the right, for each 10 years of reduction in the dataset age range. Moreover, fewer significant probes were associated with the first age-position (Figure 2), whilst the second age-position was less influenced with respect to the occurrence age and the number of probes associated with (Figure 2). In the 45–106 years dataset the first age-position was not stable due to the low sample resolution, but the second age-position was unchanged (Figure 2). To assess the effect of centenarians, another artificial dataset was generated by trimming the brain cortex dataset from the right, discarding the elderly (>87 years). Also in this dataset, two distinct age-positions were identified, but compared with the original dataset, the second age-position was more affected with respect to the occurrence age and the number of significant probes (Figure 2). Together this demonstrates that in the brain cortex dataset, two distinct age-positions where expression trends bend are stable and consistent, but the exact point is subject to variation according to the age range of the dataset.

Next, we examined the effect of the dataset resolution on the occurrence of the age-positions. Since the kidney cortex dataset contained the largest number of subjects, we compared the full dataset (N = 72) with a generated dataset that includes only half of the subjects (N = 36; Additional file 1: Figure S4). To avoid a change in the distribution, the artificial dataset was generated by removing every other sample, maintaining their distribution across decades (Additional file 1: Table S2). Age-positions did not differ between the original kidney cortex and ‘half’ of the dataset (Additional file 1: Figure S4). This indicates that the resolution in an evenly age distributed dataset has little impact on the occurrence of the age-positions.

**Figure 3:**
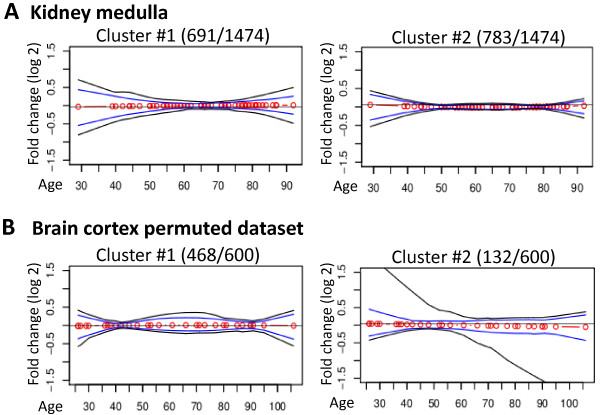
**Age-positions are not found in datasets without a significant age-regulation.** Plots show *k*-means absolute correlation clusters from kidney medulla dataset **(A)**, or from brain cortex **(B)**. The red circles connected by red lines represent the cluster centroids. The 95th percentile depicted in blue represents the major trends in the cluster. The 99th percentile shows the within cluster variation. The number of probes per cluster, as opposed to the total number of identified significant probes is denoted in the title of each plot.

The stability of the age-positions and age-associated trends was also evaluated in the kidney medulla dataset. RNA expression profiles in this dataset did not present a significant age-association (Table 1). The trends generated by the 1474 probes passing the *p*-value filtering, had a lower fold-change compared with the age-regulated datasets. Moreover, the age-positions were not clear or consistent (Figure 3A). As 1474 probes passed the filter for significant age-association, but the dataset itself did not pass under the *globaltest* threshold, it is highly possible that a considerable amount of these probes represent false-positives.

To further investigate the effect of false positive probes, the age-positions were assessed in the permuted datasets from brain cortex (Figure 3B) and kidney cortex (Additional file 1: Figure S5), where samples were not ordered according to chronological age. In these permuted datasets, age-positions were not clear and not consistent. Together this demonstrates that stable and well defined age-positions can be found in chronologically ordered, age-associated datasets, but not in datasets with insignificant age-regulation.

**Figure 4:**
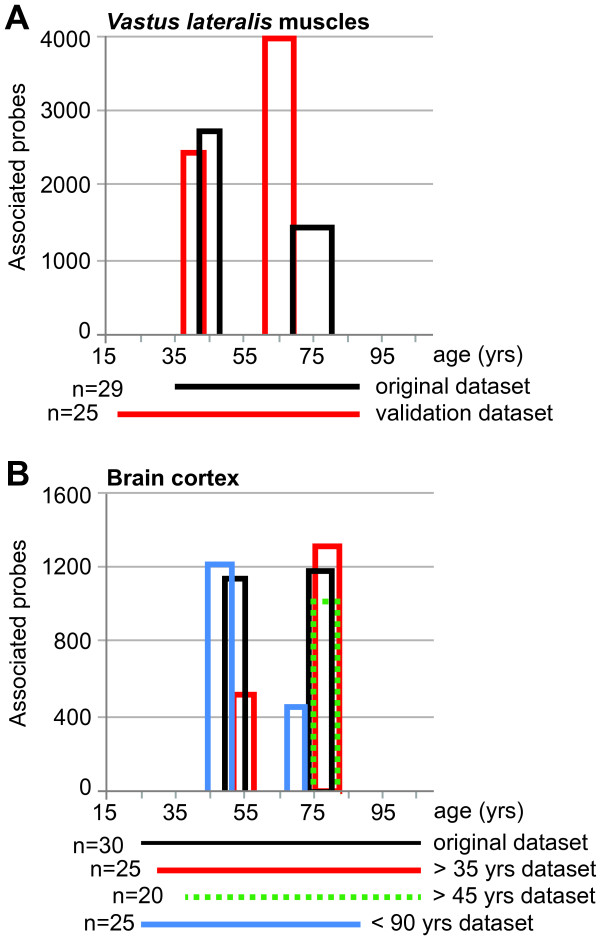
**Summary of the validation and stability of the age-positions.** Plots show temporal changes in age-positions in the validation dataset for *VL* muscles **(A)** and in brain cortex **(B)**. The age-position is denoted with the number of associated probes. Original datasets are denoted with a black line. The number of samples and the age range of every dataset is indicated under the *X*-axis. Age-positions in *VL muscles*. The age range (depicted with a line) and the number of samples per dataset is denoted under the *X*-axis. Absolute correlation *k*-means clustering plots for each dataset are shown in the Additional file 1.

To verify and validate the specificity of the age-positions and to point the limitations of the procedure, this methodology was applied on several independent and modified datasets. For validations of the age-positions in *VL* muscles we used an independent *VL* muscles dataset, where the age range is wider (17–89 years) but the number of samples is slightly smaller (N = 25) compared with the original *VL* muscles dataset (N = 29). Also in the validation dataset, two distinct age-positions were identified and these were close to the age-positions from the original dataset (Figure 4 and Additional file 1: Figure S6). As expected, the lower sample resolution in the validation dataset induces higher variation compared with the original dataset (Additional file 1: Figure S6). This suggests that age-positions do not result from technical or methodological artifacts, but represent a biological phenomenon. We noticed that in the datasets with a wider age range, the age-positions shifted about 3 years earlier compared with the original dataset (Figure 4). This suggests that the age range of the dataset could have an impact on the point at which the age-positions occur.

### Molecular composition of early and late age-positions is distinct

For molecular characterization of the two distinct age-positions, a comparison between the age-positions in *VL* muscles and brain cortex was made. The overlap of genes (Entrez ID) between the tissues at each age-position was limited (5-9%; Table 2B). However, a similarity was found in the fold-change direction: in both tissues the proportion of up-regulated and down-regulated probes was similar in the 1st age-position, but the down-regulated probes were enriched in the 2nd age-position (Table 2A). This suggests that different molecular processes regulate transcriptional changes at each age-position and those differ between tissues.

To assess the function of genes, Gene Ontology (GO) enrichment of the significant genes at each age-position was investigated using unique Entrez IDs (genes are listed in Additional file 2: Tables S4-7). Significant enrichment of GO terms was determined using Fisher’s exact test in a genome-wide context (*p* < 0.05) and subsequently, using hierarchical clustering (a complete list of the hierarchical clusters per tissue per age-position is found in Additional file 3: Tables S8-11). In agreement with the origin of the tissues, the muscle contraction and the nerve cell system were the most prominently enriched biological processes in both age-positions in *VL* muscles and in brain cortex, respectively (Table 3). The vast majority of significantly enriched GO terms differ between the two age-positions and between tissues. However, calcium transport and lipid metabolism were affected in the 1st age-position in both tissues, and in the 2nd age-position apoptosis and mitochondria (Table 3).

**Table 3:** **A summary of GO terms that are significantly enriched in 1st (early) or 2nd (late) age-positions in *VL muscles *** **or brain cortex**

	**1st age-position**	**2nd age-position**
***VL *** **muscles**	Muscle contraction;	Contractile fiber;
	**Calcium transport;**	**Mitochondria;**
	**Lipid metabolism**	**Apoptosis;**
		Estrogen receptor signaling;
		Chromatin silencing;
		Phagocytic vesicle;
		RNA processing
**Brain cortex**	Nervous system;	Nerve cell system;
	**Calcium transport;**	**Apoptosis;**
	**Lipid metabolism;**	**Mitochondria;**
	Actin cytoskeleton;	Nucleotide biosynthesis;
	Interphase;	Microtubule cytoskeleton;
	Apoptosis;	Cell migration;
	Cell migration;	Ion homeostasis;
	Nucleotide biosynthesis;	Insulin-like growth factor signaling;
	Energy biogenesis;	Hormone secretion
	Androgen signaling;	
	Protein tyrosine kinase;	
	Endocytic vesicle	

A list of significantly enriched GO terms in the early and late age-positions for *VL* muscles and brain cortex datasets. GO terms are ordered according to their significant enrichment. In bold are similar GO terms in both tissues. A complete list of the GO terms hierarchical trees can be found in Additional file 3: Tables S8–11.

## Discussion and conclusions

Declines with age in tissue and cell functionality characterize biological aging. In cross-sectional datasets obtained from healthy humans, a decline in muscle strength starts only after midlife. Using two linear models the decline is significant from the 6th decade and then after it linearly progresses [11]. More recently, we identified a decline in RNA expression of PolyA RNA binding protein 1 (PABPN1) in *VL* muscles from the fifth decade [9]. In agreement with these studies, we found that in *VL* skeletal muscles a major change in expression trends occurs during early midlife. In addition, we identified a second age, around 70 years, where expression trends changed. Coinciding with the 2nd age-position, the muscle waste also known as sarcopenia, is common in elderly [12].

Two distinct age-positions were also found in the brain frontal cortex. Our analysis demonstrated that the age-positions are consistently found in age-regulated datasets, as compared with the non-age-regulated datasets. The exact age at which trends change is significantly affected by the age-range of the dataset. A shift of ~3 years is to be considered based on the age range of the dataset (Figure 4B). In contrast, in the kidney cortex only one age-position was identified. This suggests that in humans, tissue aging differs by temporal and spatial means. As a support, very little gene overlap was found between tissues. Moreover, on the functional groups level, only two groups were similar in brain cortex and *VL* muscles. This suggests that aging in humans is not a linear process, but progresses through at least two age-positions in skeletal muscle and brain tissue. Analysis of the brain cortex dataset suggested qualitative changes around age 40 and around age 70. Our findings are consistent with [8], but provide a quantitative description of temporal changes during aging. We applied observant tests and in-depth analyses, which allow us to conclude that the age-positions identified with our methodology are stable and are not platform-dependent. Albeit only limited similarity was found between the four tissues presented in this study, as a proof of concept, conclusions regarding the differences between tissues during aging should be made with a larger comparative study. In addition, during aging, cell composition within a tissue can also change. This study, however does not address possible age-regulated changes in cell composition within a tissue.

Based on the datasets used here, the 1st age-position in skeletal muscles is around 40 years of age whereas in brain it occurs around 50 years of age. Since the sample resolution and age-range in both datasets is comparable, it suggests that expression changes in skeletal muscles occur earlier compared with the brain cortex. Although additional studies are required to confirm this observation, it may have an impact on approaches to improve healthy aging. The 2nd age-position in both datasets is found around 70 years of age and it is characterized by an enrichment of down-regulated genes, that cluster into well known aging-regulated processes [3] such as: apoptosis, mitochondria, hormonal signaling pathways and could mark tissue degeneration. The 1st age-position in both tissues is enriched with changes in calcium homeostasis and lipid metabolism. Calcium homeostasis is essential for the maintenance of muscle and nerve cells, and physiological implications have been demonstrated [13,14]. The molecular regulation of calcium homeostasis to cellular aging is not fully understood and requires additional investigation. The impact of lipid metabolisms during aging was studied in more detail, as adiposity increases in a number of tissues [15]. Recent studies in centenarian cohorts identified regulators of lipid metabolism as candidates for longevity in humans [16,17]. From the list of genes (Additional file 2: Tables S4–7), in each age-position additional regulators of aging in humans could be identified for functional studies.

A genome-wide decline in gene expression in the 2nd age-position could contribute to tissue degeneration in elderly. To assess the impact of the age-positions on longevity, we have analyzed a ‘trimmed’ dataset, excluding samples above 87 years of age. Although two age-positions were found in this analysis, we argue that we cannot address the impact of the late age-position to longevity. Due to the nature of cross-sectional studies, like those used here, the late age-position could also present survivor effects of those individuals. For instance, it may be possible to observe an expression change in the population above age 90, as they become part of a more exclusive population. Changes in expression may occur either due to level increases in every individual at 90 years of age, or due to the individuals with low expression that have small probability of survival until this age and above. In other words, it is also possible that cross-sectional datasets reveal the enrichment in survivor populations. Only longitudinal studies following the same subjects in time will be able to make a distinction between these two possibilities and to reveal an impact on longevity.

In statistical modeling there are two competing issues at stake. The first is avoiding bias that arises from the model assumptions that may not be true. The second is avoiding variance that arises from a model that is too flexible, thus the model would overfit the data. Previous studies in aging microarray datasets applied linear models in cross-sectional datasets [6,7,18]. Linear models, however, make many assumptions, which are doubtful in complex biological processes. It is highly unlikely that trends in biology are exactly linear. Here we have carefully compared linear, quadratic and cubic models on three different chronologically ordered datasets and found that the quadratic and cubic models outperformed the linear model. Although we chose to apply a quadratic regression model in our studies here, we recognize that more complex models could be investigated in future studies. The datasets used here are not dense enough for such models, thus the use of a dataset with multiple samples per year and more uniformly distributed samples across age is advised.

## Methods

The inclusion criteria for this study are: even data distribution in every decade (at minimum starting from 40 years) with a minimum of 25 samples (Additional file 1: Table S1). In order to minimize the loss of information, analyses were carried out on probe ID, covering from 48826 to 12567 unique probes per dataset, depending on the platform used. Subsequently, an age-regulation of mRNA profiles in each of the datasets was determined using the *globaltest*[19] where the *p*-value calculation assumes no-age association as the null hypothesis. Gender correction was also included into the *globaltest* model.

### Microarray datasets

The dataset from *VL* muscles was generated from healthy humans not presenting a chronic disease. All muscle biopsies were collected using the Bergstrom needle at Canisius-Wilhelmina Hospital, Nijmegen, The Netherlands, after an approval of the medical ethical committee Arnhem-Nijmegen (CMO nr. 2005/189) and a written informed consent from participants, as described in [20]. Biopsies were immediately frozen in liquid nitrogen and stored at -80°C before RNA extraction. RNA extraction, labeling and hybridization on Illumina Human v3.1 arrays were performed as described in [20]. Our muscle dataset is deposited for public use (GEO-GSE40645). The microarray datasets in this study was retrieved from brain frontal cortex: (GEO-GSE1572) [8], kidney cortex and kidney medulla, obtained from Stanford Microarray Dataset (http://cmgm.stanford.edu/~kimlab/aging_kidney/). In the *Rectus abdominis* dataset, 19 samples from other muscles (*Deltoid, Arm muscle, Biceps, Quadriceps, Fascia, Thigh muscle, Gastrocnemius, upper arm muscle)* were excluded from this analysis. A second dataset from *VL* muscles (referred here as B) can be found at (GSE26605) [21]. Platforms of all datasets are detailed in Additional file 1: Table S1.

### Pre-processing

All datasets were pre-processed with a uniform protocol including: variance stabilization and normalization (*vsn* v3.20.0 [21]), *limma* v3.8.3 [22], *lumi* v2.4.0 [23], *affy* v1.30 [24], and *affydata* v1.13.11 packages in R), as well as batch correction (*ComBat* R script available at http://www.bu.edu/jlab/wp-assets/ComBat/Abstract.html) for the *VL* muscles dataset. The principal component analysis (PCA), implemented in the *princomp* function in R package was applied in order to identify potential outliers present in the dataset. Eventually, all samples were included in the study. In the kidney datasets, duplicates as well as individuals without gender assignments were excluded (Additional file 1: Table S1). A significant age-association (*p*-value < 0.05) for each dataset was determined after gender correction using the *globaltest* (*globaltest* v5.10.0 package in R) [25].

### Probe smoothing

To identify age-related trends, smoothing was carried out per probe for each of the available tissues. Several smoothing models were considered. Spline functions [26] (including: cubic spline, Bézier curves, quadratic B-spline) and a local regression method (LOESS) [26] were compared with a simple linear regression (examples are shown in Additional file 1: Figure S1A). The performance of the smoothing methods was statistically evaluated using ‘leave-one-out’ cross-validation (LOOCV) with a *k*-fold equal to the dataset resolution. The fitted values, as well as the residuals were computed for all probes in the *VL* muscles dataset and the average sum of squares error represents the fitness measure of each smoothing method (Additional file 1: Figure S1B). A detailed presentation of each model is found in the description of Additional file 1: Figure S1. Probe smoothing was separately applied on each dataset. An overlap of the age-regulated genes between tissues was assessed using unique Entrez IDs (Additional file 1: Table S3).

The quadratic curve was chosen over the higher order and more complex spline models, as these showed a tendency to overfit the data due to the larger number of parameters in the model. The LOESS function was discarded due to the visible influence of a “*wiggle”* effect, as the LOESS allows more curvature in denser age neighborhoods. An example of this effect is shown in Additional file 1: Figure S1A. The absence of control points for the fitting curve of the quadratic B-spline function is adequate for an unbiased analysis and the use of a quadratic model allows more freedom in expression trend identification.

### Probe filtering

Age-regulated probes were filtered based on our quadratic model, testing the null hypothesis of no age association *versus* the quadratic age model described above. Only the probes resulting in a *p-*value < 0.05 (unadjusted) were kept for further analysis. Examples of significant and non-significant probes are shown in (Additional file 1: Figure S2).

### Clustering

Prior to clustering, the smoothened values of the filtered probes per dataset were normalized by subtracting the average expression value of a probe from each of its data points. This allows clustering based on trend similarity rather than expression values. The clustering was performed using *k-*means with Euclidean distance as metric, aiming at minimizing the sum of squares error between the data points of a probe and the designated cluster centroids. The cluster centroids were randomly initialized by the internal mechanisms of the clustering functions, in order to respect the unbiased nature of the study. Their final position is obtained once the algorithm converges, based on the procedure in [27]. The resulting clusters were evaluated for confidence with the 99th and 95th percentile. The 95th percentile shows the borders of the dominant trends in a cluster and the variations within each trend were evaluated by point-wise reference intervals at the 95th and 99th percentile (e.g., Figure 1, Additional file 1: Figure S3B). The stability of the clusters was evaluated by cycles of re-clustering from 24, 16, 12, 8, 6 and 4 clusters until obtaining a minimum number of consistent clusters defining the major expression trends. Cluster stability was assessed with the sum of squares error between the centroids of a cluster and its associated probes. A genetic version of the *k*-means algorithm was used [28] to evaluate the robustness of the classic *k*-means algorithm convergence (Additional file 1: Figure S3A). Using the classic *k*-means algorithm, implemented in the R base package *cluster*, for N = 4 clusters, 70% of the runs converged exactly to the same point. This provides a rough estimation for the minimum and most stable number of clusters in this analysis. A horizontal symmetry of the trends was evaluated with absolute correlation as a distance metric in the clustering algorithm, using the *Kmeans* function in the R package *amap*. Age-positions were estimated from the intersection of symmetric trends within an absolute correlation cluster, where the 95th percentile is closer to the centroids. The age range of an age-position was determined from the *k-*means absolute correlation clustering in 2, 3 and 4 clusters.

*Functional analysis* was carried out with Entrez IDs. Probes were annotated per platform and were mapped to their corresponding Entrez ID, using the R packages *lluminaHumanv3BeadID* for *VL* muscles, *hgu95av2.db* for the brain frontal cortex and *hgu133a.db* for the kidney dataset. A small percentage of the data was lost, as not all probes are annotated and not all annotated probes have an Entrez ID (Additional file 1: Table S1). The unique genes forming the absolute correlation clusters were mapped cluster-wise onto the Gene Ontology (GO) database using the *org.HS.eg.db* database from Bioconductor. The significant GO terms were selected using Fisher’s exact test in R (*p*-value < 0.05). The GO term enrichment was calculated using as background the entire mapping to Entrez IDs per platform. In the brain cortex, the GO term *p*-values were adjusted for multiple testing using the false discovery rate procedure [29]. Subsequently, redundant GO terms were removed. Generic (>1000 genes) as well as to specific (<10 genes) terms were discarded before the following step. The significant GO terms were grouped into hierarchical functional clusters using the GO database structure, which allows the search for interrelated GO terms and, moreover, provides information about their degree of relation.

### Availability of supporting data

Our microarray data from VL muscles is deposited as (GEO-GSE40645) and publically available. Genes from first and second age-positions are available in text format in Additional file 2: Tables S4–7 and hierarchical trees of the significant GO terms from first and second age-positions are found in Additional file 3: Tables S8–11.

## Competing interests

The authors declare that they have no competing interests.

## Authors’ contributions

MG carried out all componential, statistical and bioinformatic analysis, as well as drafting the manuscript and supplementary file. MS collected muscle biopsies. ME and TB helped in design of componential analysis. JJG supervised statistical analysis. VR designed and coordinated the study and contributed to the writing of the manuscript. All authors read and approved the final manuscript.

## Supplementary Material

Additional file 1: Figure S1 A comparison of data smoothing methods. **Figure S2.** An example of expression trends in a significant or an insignificant probe. **Figure S3.***K*-means clustering using Euclidean distance applied to the significant probes of the *Vastus lateralis* dataset. **Figure S4.** The effect of the dataset size and resolution on age-positions. **Figure S5.** Age-positions are not consistent in permuted datasets. **Figure S6.** Absolute correlation *k-*means clustering applied on the significant probes in *Vastus lateralis.***Table S1.** A summary of the platform details of the datasets that were used in our study. **Table S2.** Age distribution per decade in four datasets that were used for trend analysis. **Table S3.** Overlap of age-associated Entrez ID between tissues. Click here for file

Additional file 2: Tables S4-7 Gene lists of Entrez ID and their gene symbols, for genes that are associated with the early (Tables S4 and S5) or late **(Tables S6 and S7)** age position in Brain cortex **(Tables S4 and S6)** or *VL* muscles **(Tables S5 and S7)**, as well as lists of the overlapping genes per age-position. Click here for file

Additional file 3: Tables S8-11 Hierarchical clustering per tissue and per age-position of the significant GO terms. Click here for file

## References

[B1] Vijg J Suh Y Genetics of longevity and aging Annu Rev Med 2005 56 193 212 10.1146/annurev.med.56.082103.104617 15660509

[B2] Wheeler HE Kim SK Genetics and genomics of human ageing Philos Trans R Soc Lond B Biol Sci 2011 366 1561 43 50 10.1098/rstb.2010.0259 21115529 PMC3001305

[B3] Passtoors WM Beekman M Gunn D Boer JM Heijmans BT Westendorp RG Zwaan BJ Slagboom PE Genomic studies in ageing research: the need to integrate genetic and gene expression approaches J Intern Med 2008 263 2 153 166 10.1111/j.1365-2796.2007.01904.x 18226093

[B4] Erraji-Benchekroun L Underwood MD Arango V Galfalvy H Pavlidis P Smyrniotopoulos P Mann JJ Sibille E Molecular aging in human prefrontal cortex is selective and continuous throughout adult life Biol Psychiatry 2005 57 5 549 558 10.1016/j.biopsych.2004.10.034 15737671

[B5] Zhan M Yamaza H Sun Y Sinclair J Li H Zou S Temporal and spatial transcriptional profiles of aging in Drosophila melanogaster Genome Res 2007 17 8 1236 1243 10.1101/gr.6216607 17623811 PMC1933522

[B6] Rodwell GE Sonu R Zahn JM Lund J Wilhelmy J Wang L Xiao W Mindrinos M Crane E Segal E A transcriptional profile of aging in the human kidney PLoS Biol 2004 2 12 e427 10.1371/journal.pbio.0020427 15562319 PMC532391

[B7] Passtoors WM Boer JM Goeman JJ Akker EB Deelen J Zwaan BJ Scarborough A Breggen R Vossen RH Houwing-Duistermaat JJ Transcriptional profiling of human familial longevity indicates a role for ASF1A and IL7R PLoS One 2012 7 1 e27759 10.1371/journal.pone.0027759 22247756 PMC3256132

[B8] Lu T Pan Y Kao S-Y Li C Kohane I Chan J Yankner BA Gene regulation and DNA damage in the ageing human brain Nature 2004 429 6994 883 891 10.1038/nature02661 15190254

[B9] Anvar SY Raz Y Verway N van der Sluijs B Venema A Goeman JJ Vissing J van der Maarel SM t Hoen PA van Engelen BG A decline in PABPN1 induces progressive muscle weakness in Oculopharyngeal muscle dystrophy and in muscle aging Aging (Albany NY) 2013 5 6 412 426 23793615 10.18632/aging.100567 PMC3824410

[B10] Tusher VG Tibshirani R Chu G Significance analysis of microarrays applied to the ionizing radiation response Proc Natl Acad Sci U S A 2001 98 9 5116 5121 10.1073/pnas.091062498 11309499 PMC33173

[B11] Beenakker KG Ling CH Meskers CG de Craen AJ Stijnen T Westendorp RG Maier AB Patterns of muscle strength loss with age in the general population and patients with a chronic inflammatory state Ageing Res Rev 2010 9 4 431 436 10.1016/j.arr.2010.05.005 20553969 PMC7105185

[B12] Goodpaster BH Park SW Harris TB Kritchevsky SB Nevitt M Schwartz AV Simonsick EM Tylavsky FA Visser M Newman AB The loss of skeletal muscle strength, mass, and quality in older adults: the health, aging and body composition study J Gerontol A Biol Sci Med Sci 2006 61 10 1059 1064 10.1093/gerona/61.10.1059 17077199

[B13] Weisleder N Ma JJ Ca2+ sparks as a plastic signal for skeletal muscle health, aging, and dystrophy Acta Pharmacol Sin 2006 27 7 791 798 10.1111/j.1745-7254.2006.00384.x 16787561

[B14] Foster TC Kumar A Calcium dysregulation in the aging brain Neuroscientist 2002 8 4 297 301 10.1177/107385840200800404 12194497

[B15] Harris TB Invited commentary: body composition in studies of aging: New opportunities to better understand health risks associated with weight Am J Epidemiol 2002 156 2 122 124 10.1093/aje/kwf024 12117701

[B16] Vaarhorst AA Beekman M Suchiman EH van Heemst D Houwing-Duistermaat JJ Westendorp RG Slagboom PE Heijmans BT Lipid metabolism in long-lived families: the Leiden longevity study Age 2011 33 2 219 227 10.1007/s11357-010-9172-6 20811950 PMC3127468

[B17] Barzilai N AGSC UNique lipoprotein phenotype and genotype associated with exceptional longevity JAMA 2003 290 15 2030 2040 10.1001/jama.290.15.2030 14559957

[B18] Zahn JM Sonu R Vogel H Crane E Mazan-Mamczarz K Rabkin R Davis RW Becker KG Owen AB Kim SK Transcriptional profiling of aging in human muscle reveals a common aging signature PLoS Genet 2006 2 7 e115 10.1371/journal.pgen.0020115 16789832 PMC1513263

[B19] Goeman JJ van de Geer SA de Kort F van Houwelingen HC A global test for groups of genes: testing association with a clinical outcome Bioinformatics 2004 20 1 93 99 10.1093/bioinformatics/btg382 14693814

[B20] Anvar SY t Hoen PA Venema A van der Sluijs B van Engelen B Snoeck M Vissing J Trollet C Dickson G Chartier A Deregulation of the ubiquitin-proteasome system is the predominant molecular pathology in OPMD animal models and patients Skeletal Muscle 2011 1 1 15 10.1186/2044-5040-1-15 21798095 PMC3156638

[B21] Huber W von Heydebreck A Sultmann H Poustka A Vingron M Variance stabilization applied to microarray data calibration and to the quantification of differential expression Bioinformatics 2002 18 Suppl 1 S96 S104 10.1093/bioinformatics/18.suppl_1.S96 12169536

[B22] Smyth GK Linear models and empirical bayes methods for assessing differential expression in microarray experiments Stat Appl Genet Mol Biol 2004 3 12 10.2202/1544-6115.1027 16646809

[B23] Du P Kibbe WA Lin SM lumi: a pipeline for processing Illumina microarray Bioinformatics 2008 24 13 1547 1548 10.1093/bioinformatics/btn224 18467348

[B24] Gautier L Cope L Bolstad BM Irizarry RA affy-analysis of Affymetrix GeneChip data at the probe level Bioinformatics 2004 20 3 307 315 10.1093/bioinformatics/btg405 14960456

[B25] Fan J Yao Q Nonlinear Time Series: nonparametric and parametric methods 2005 New York: Springer

[B26] Cleveland WS Grosse E Computational methods for local regression Stat Comput 1991 1 1 47 62 10.1007/BF01890836

[B27] Hartigan JA Wong MA Algorithm AS 136: A K-Means Clustering Algorithm J R Stat Soc Series C (Applied Statistics) 1979 28 1 100 108

[B28] Krishna K Narasimha Murty M Genetic K-means algorithm IEEE Trans Syst Man Cybern B Cybern 1999 29 3 433 439 10.1109/3477.764879 18252317

[B29] Benjamini Y Hochberg Y Controlling the false discovery rate: a practical and powerful approach to multiple testing J R Stat Soc Series B (Methodological) 1995 57 1 289 300

