# Supplementary material for: Major aging-associated RNA expressions change at two distinct age-positions
Source: BMC Genomics. 2014 Feb 14;15:132. doi: 10.1186/1471-2164-15-132 (PMC3930826; doi:10.1186/1471-2164-15-132)
Supplement: Additional file 1: Figure S1 — A comparison of data smoothing methods. Figure S2. An example of expression trends in a significant or an insignificant probe. Figure S3.K-means clustering using Euclidean distance applied to the significant probes of the Vastus lateralis dataset. Figure S4. The effect of the dataset size and resolution on age-positions. Figure S5. Age-positions are not consistent in permuted datasets. Figure S6. Absolute correlation k-means clustering applied on the significant probes in Vastus lateralis.Table S1. A summary of the platform details of the datasets that were used in our study. Table S2. Age distribution per decade in four datasets that were used for trend analysis. Table S3. Overlap of age-associated Entrez ID between tissues. [file 1471-2164-15-132-S1.docx]

**SUPPLEMENTARY FILE:**

**SUPPLEMENTARY FIGURE 1: A comparison of data smoothing methods**


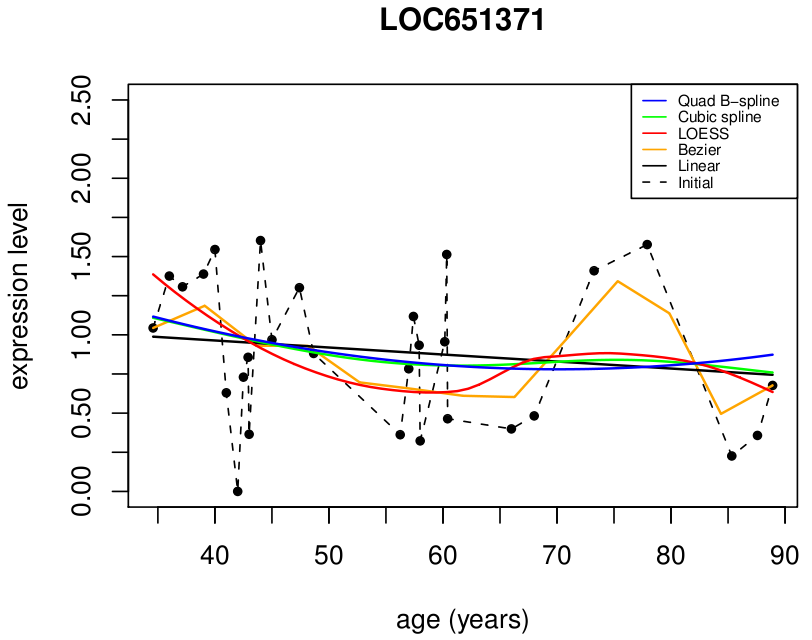

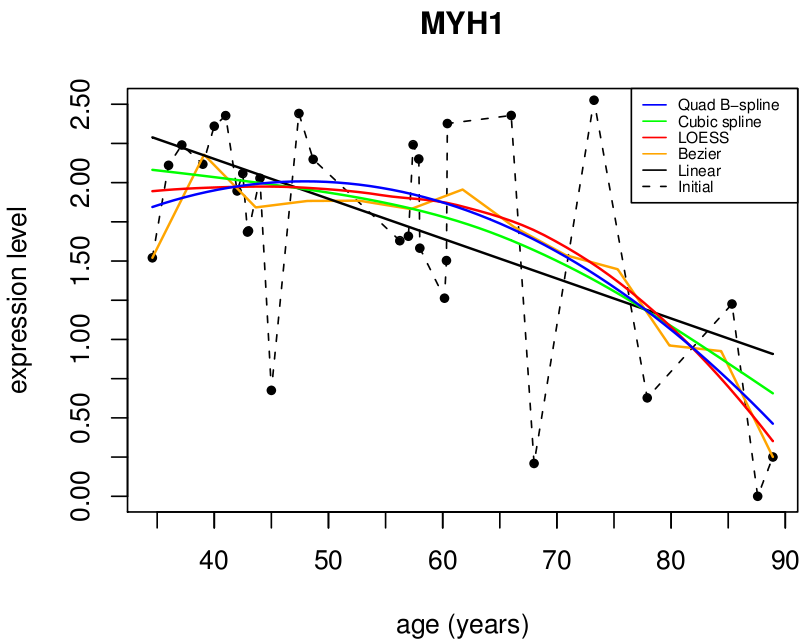


**A**


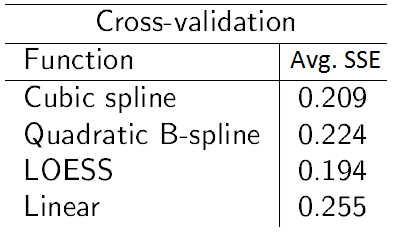

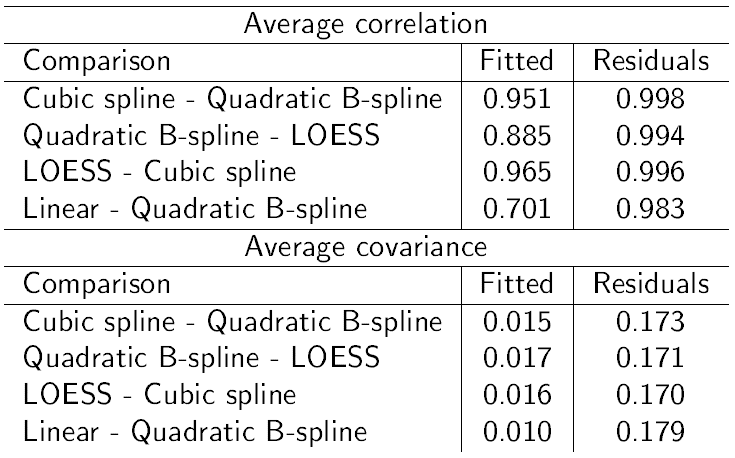


**B**

**A.** Plots show five data smoothing methods applied on a random gene (left) and a known age-regulated gene (right) from the *Vastus lateralis* dataset. The initial data points are depicted by filled black circles interconnected by a dashed line. A linear regression is shown with a black line. Bézier curves, which are a series of splines, are depicted in orange. Twelve control points (or knots) were used in the generation of the Bézier fitting curve, which help to reduce angularity. Increasing the number of knots beyond the aforementioned value did not affect the fitting curve. Characteristic of the Bézier curves is that the fitting curve starts and ends from the first and last data points and the distribution of the samples has a major impact on the resulting fitting curve. The local regression (LOESS) fitting curve is shown in a red line. LOESS, as opposed to LOWESS (locally weighted scatter plot smoothing), uses quadratic interpolation instead of linear interpolation and does not require a fitting function, thus no theoretical background about the model is needed. The use of quadratic interpolation allows the identification of both non-linear and linear expression trends. The size of the neighborhood used in the predictor of the curve is equal to the sample size, thus all the data points are considered of equal significance. The center of the neighborhood influences the shape of the fitting curve and can produce a *wiggle* effect (left plot). The sample distribution directly affects the intensity of the *wiggle* effect. A cubic spline, shown in the green line, uses a third-order polynomial to produce a sufficiently smooth fitting curve. The control knots of the fitting curve are equally distributed along the sample size. The third degree polynomial used in the smoothing function shows a tendency to overfit the data, as the resulting curve depends on the data distribution and the amplitude of the control points. The quadratic B-spline, depicted in blue, is a generalization of Bézier curves. The basis of the spline uses a quadratic term in the regression model, allowing non-linear trends to be identified, as well as the linear ones, without overfitting the data. No control points are used in the generation of the fitting curve, thus the data distribution does not affect the behaviour of the function and thus it is suitable when applied to datasets with no prior knowledge of the data. In the context of an unbiased gene expression study, it allows the comparison of profile trends between different datasets.

**B.** Tables show the results of comparative statistical tests between the regression models using higher order polynomials and between the preferred quadratic model and linear regression in *vastus lateralis* muscles. Each test was performed on the fitted values, as well as on the residual values of each regression model. A residual value represents the sum of squared errors between the predicted point and the initial data point. The results are averaged over all the probes in the *Vastus lateralis* dataset (e.g., 48803). The correlation value indicates that these three smoothing methods are highly similar. The value of the covariance denotes the difference between the internal mechanisms of the regression models. `Leave-one-out’ cross-validation, which determines how well the fitting curve predicts a missing data point, was applied with a *k*-fold equal to the population size, in order to test the robustness and efficiency of the methods. The average sum of squared errors (SSE) of the fitting curves generated during the cross-validation was calculated per probe and averaged overall the dataset. It shows that the smoothing methods are robust and behave similar at predicting missing points. The linear model though behaves less good than the models using higher order polynomials.

A spline function is a polynomial function, which passes through a defined set of control points or knots. For our analysis, spline interpolation is preferred over simple polynomial interpolation, minimizing the interpolation error by using low degree polynomials for the creation of the smoothing function. For instance, quadratic splines use second-order polynomials to produce a sufficiently smooth fitting curve, whilst cubic splines use third-order polynomials. Basis splines or B-splines use a regression model in which the response vector is modelled by a user-defined basis of the spline function. The fitting curve in the spline function is adjusted by control points that are defined by the user and are data-dependent. The control points determining the fitting curve significantly affect the behaviour of the smoothing curve. The choice of the control points generally assumes prior knowledge of the data. LOESS is a variant of the locally weighted scatter-plot smoothing (LOWESS) for which all the elements of the weight vector influencing the response vector used in the regression model are equal. In LOESS, the interpolation is achieved by using low degree polynomials, fitted to a subset of the data. In our analysis, second-degree polynomials were used and the interpolation subset is equal to the population size. The comparison between the five candidate non-linear data smoothing methods indicates that a quadratic spline, a cubic spline and LOESS produce similar results when applied on gene expression data, with little variations due to data distribution (examples are in Supplementary Figure 1A). The linear model was also included in the statistical tests, as an indicator. As the cross validation results show (Supplementary Figure 1B), the prediction efficiency of the linear model was not as good as the higher order regression models. The covariance and correlation of the fitted and residual values of the higher order regression models, as well as the cross validation indicate no considerable difference between the quadratic spline, cubic spline and LOESS (Supplementary Figure 1B). Between functions with nearly equal fit, we preferred using a smoothing function with minimal user influence (e.g., no predefined control points or weight vectors), in order to produce an unbiased fitting curve. Based on these considerations, we chose a quadratic spline with no internal knots (i.e., a simple quadratic curve) for all datasets. More complex curves could potentially be preferable for other datasets with larger sample size.

The fitting curve we have applied is defined by the following quadratic age model:


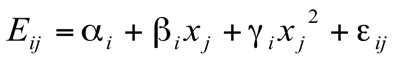


where *E_ij_* is the probe intensity i for subject *j*, *x_j_* being the age of subject *j*, α*_i_*, β*_i_*, γ*_i_* are probe-specific regression parameters, and ε*_ij_* is the residual error.

**SUPPLEMENTARY FIGURE 2 : An example of expression trends in a significant or an insignificant probe**


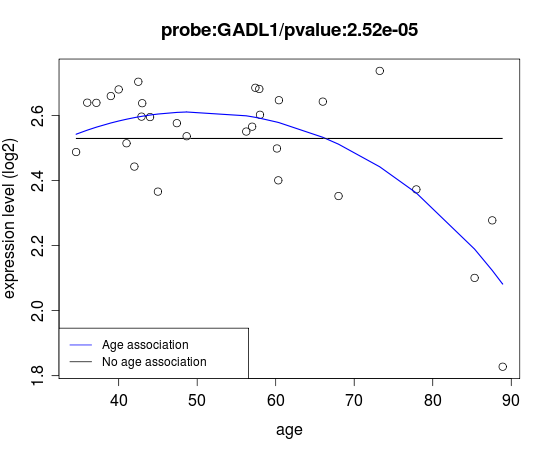

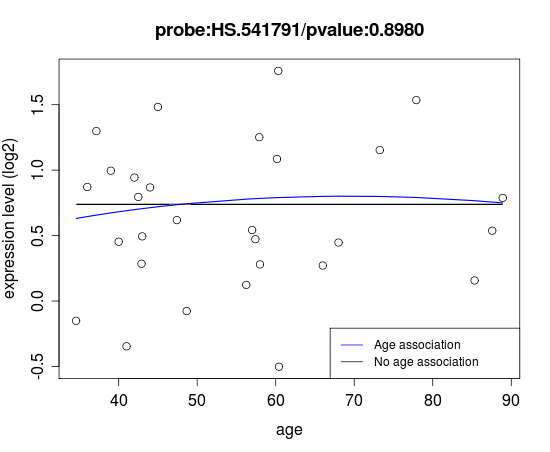


Plots show the trend of a *significant probe* (p<0.05) (left) and an insignificant one (p>0.05) (right). The empty circles denote the initial data points. The blue line represents the quadratic B-spline fitting curve. A linear regression model, assuming no age-association, is depicted with a black line.

**SUPPLEMENTARY FIGURE 3 : *K*-means clustering using Euclidean distance applied to the significant probes of the *Vastus lateralis* dataset**


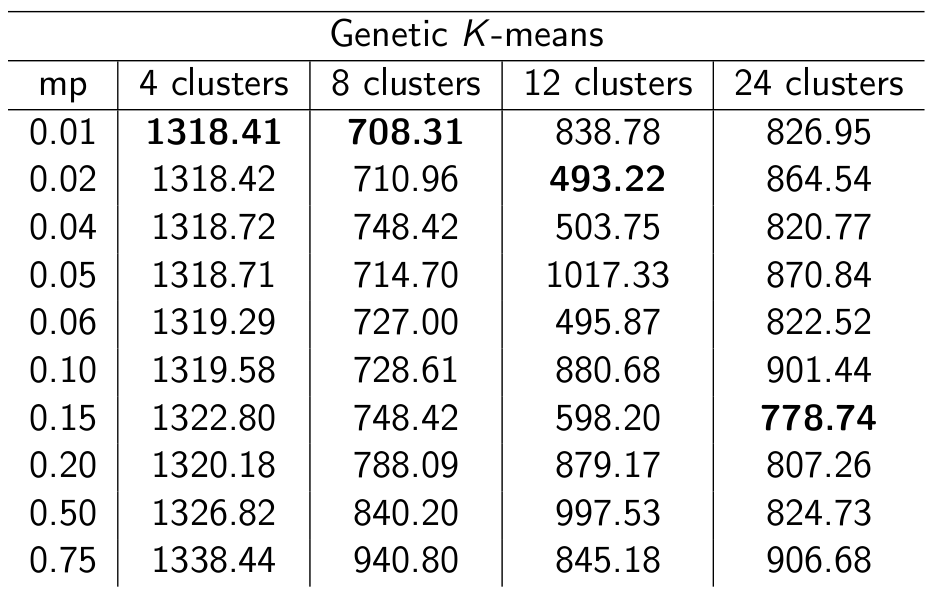

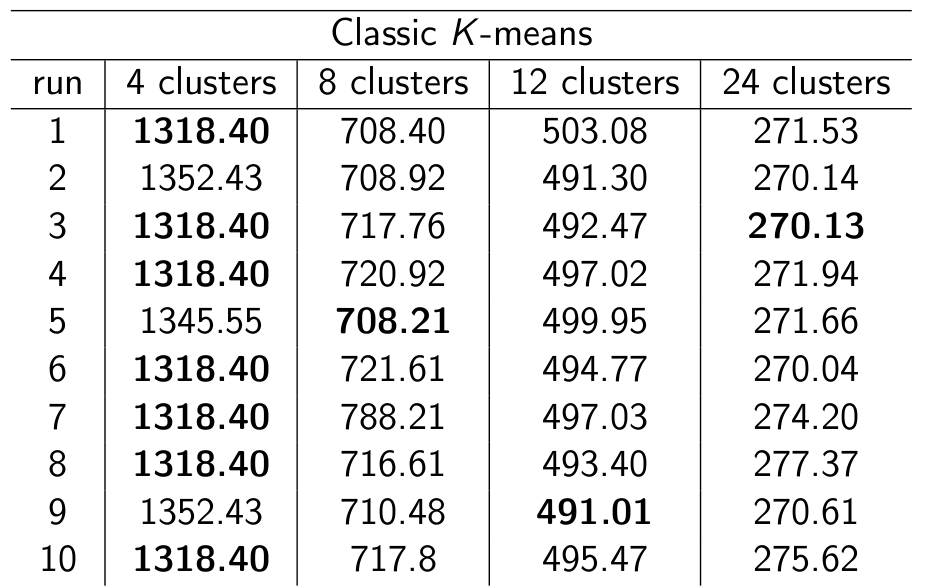


**A**


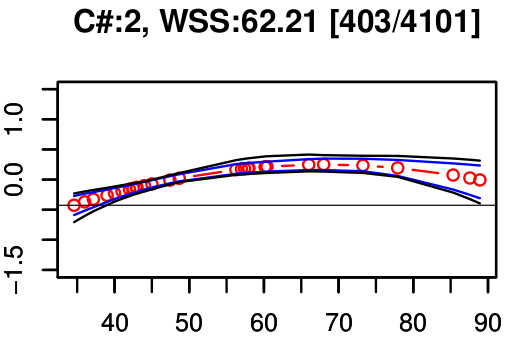


**B**


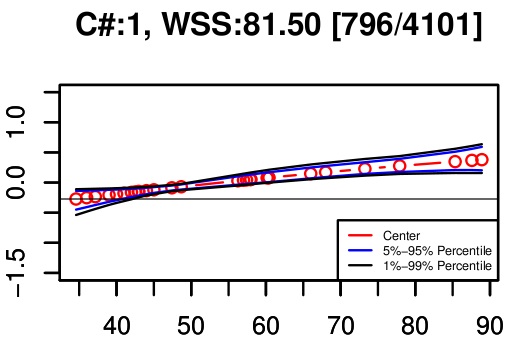

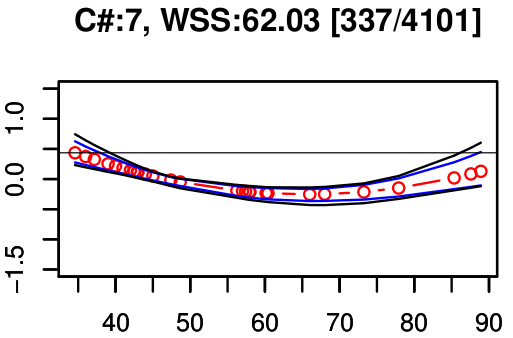

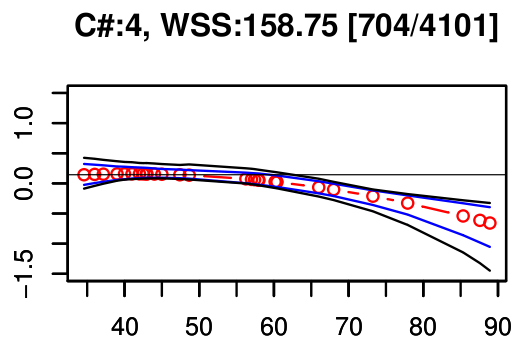

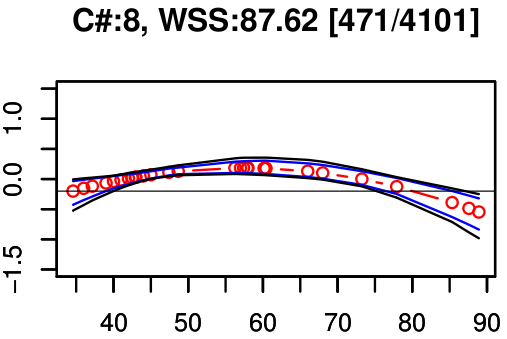


Age

Age

Age

Age


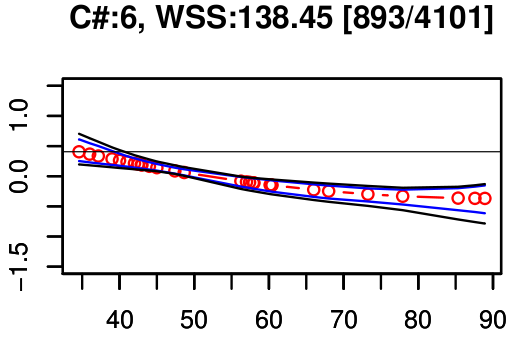


Fold change (log2)


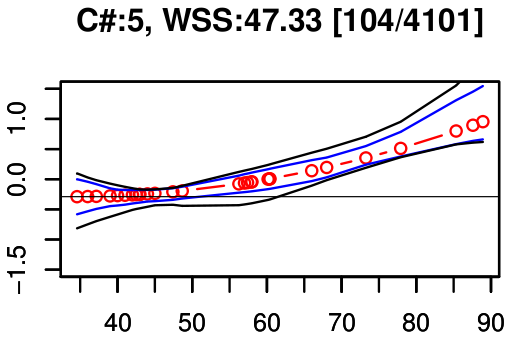


Age

Age

Age

Age


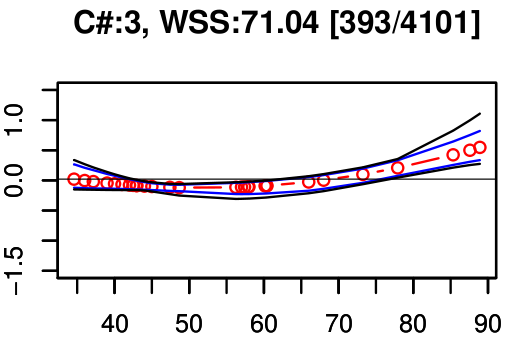


Fold change (log2)

Fold change (log2)

Fold change (log2)

Fold change (log2)

Fold change (log2)

Fold change (log2)

Fold change (log2)


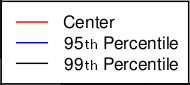


**A.** Tables show the overall sum of squares error between the cluster centroids (mean) and their associated probes, as a statistical evaluation of the robustness of the *k*-means clustering. The *k*-means clustering algorithm was applied to the significant probes, shown are the results for the *Vastus lateralis* dataset (e.g., 4101 probes). The Euclidean distance was used as a metric measure. *K*-means clustering aims at partitioning the dataset into *k* partitions such that the within sum of squared errors for each cluster is minimized. The classic *k*-means clustering algorithm is a heuristic algorithm, thus subject to returning local optima instead of the global. To verify the convergence point of the algorithm, a genetic *k*-means algorithm (GKA) [[1](#_ENREF_1)] (right) was applied in this study. Here we have used a faster variant of the GKA developed by Krishna [[1](#_ENREF_1)] entitled FGKA (fast genetic *k-*means algorithm) [[2](#_ENREF_2)]. The motivation behind this choice is the characteristic of genetic algorithms to provide the globally optimal solution in an optimization context with the right parameter settings. Here, the optimization problem is the minimization of the within cluster variation, denoted by the aforementioned sum of squared errors measure. The significant probes in the *Vastus lateralis* dataset were partitioned in 4, 8, 12 and 24 clusters respectively. Ten random starts were used while applying the classic *k*-means algorithm and the within sum of squares over all the clusters was computed for each choice of *k*. For the genetic *k*-means, different mutation probabilities (mp) were tested to determine which value provides the best solution. This is a drawback of the genetic algorithm, as the mutation probability differs for each choice of *k* and for each dataset. Moreover, the classic *k-*means algorithm, implemented in the *kmeans* function in R base package *cluster*, proved to be returning better solutions than FGKA in all configurations. The best solutions are indicated in bold. The choice of the number of clusters is a known issue when using *k*-means clustering. The number of clusters and the variations within a cluster are interrelated, thus a trade-off has to be made. This analysis suggests that *k* equal to 4 produces the most stable results.

**B.** Plots show the resulting eight clusters of significant probes in the *Vastus lateralis* dataset using *k-*means clustering with Euclidean distance as metric. The title of each plot contains the number of the cluster, the within sum of squared errors (WSS) per cluster and the number of associated probes over the total number of probes subjected to clustering. The cluster centroids are represented by the hollow red circles interconnected with a red line. The 95th percentile, in blue, denotes the confidence interval of each cluster, as well as the dominant trend, while the 99th percentile, illustrated in black, represents the trend variation within a cluster. The horizontal grey line represents a linear regression model, assuming no age-association.

**SUPPLEMENTARY FIGURE 4: The effect of the dataset size and resolution on age-positions**


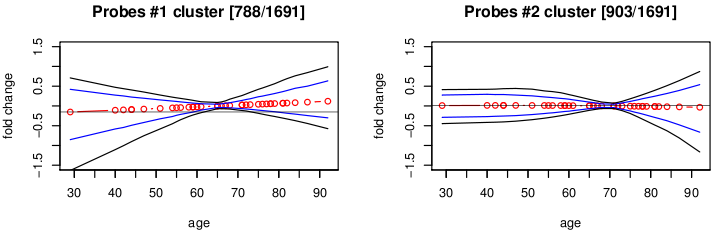

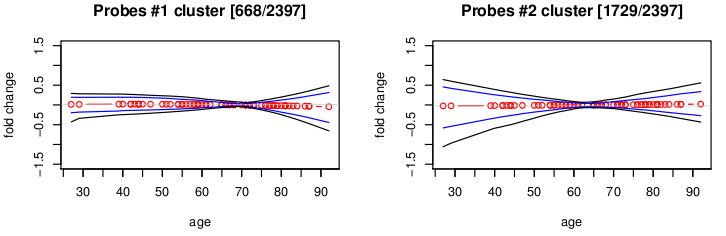

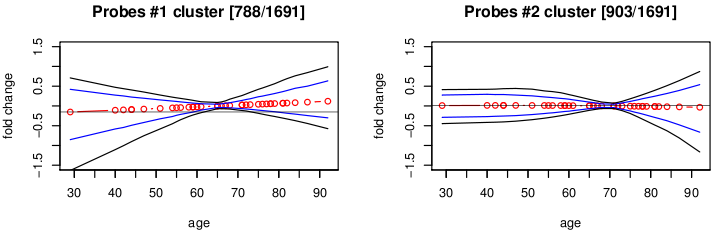

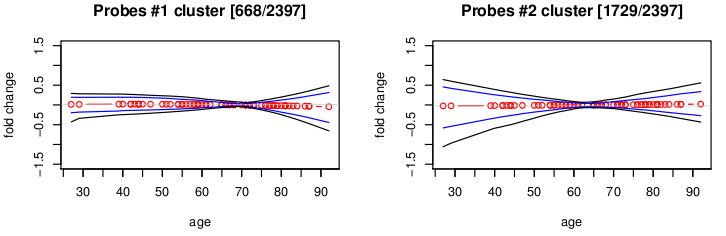


**Cluster #1 [1729 / 2397]**

**Cluster #1 [788 / 1691]**

Age

Fold change (log2)

1^st^ Age-position

**Cluster #2 [668 / 2397]**

Entire dataset

Fold change (log2)

Age

Fold change (log2)

**Cluster #2 [903 / 1691]**

2^nd^ Age-position

Half of dataset

Age

Fold change (log2)

Age


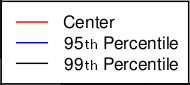


In order to determine the effect of the number of samples upon the age-positions, the entire kidney cortex dataset was compared with a variant dataset reduced in sample resolution. The variant was obtained by discarding every other sample from the original dataset, thus reducing its resolution to half. Significant probes in both datasets were subject to absolute correlation *k*-means clustering. Plots show the resulting clusters in the full kidney cortex dataset (up) and a subset of the dataset (down). The red circles interconnected with red lines represent the cluster centroids. In red, the 95th percentile denotes the dominant trends in the cluster and the 99th percentile in black shows the within cluster variation. Reducing the resolution of the dataset did not influence the occurrence of the age-positions in expression profile, but had an impact on the within cluster variation, as depicted by the 99th percentile.

For each variant dataset the age-regulated probes were clustered using absolute correlation *k-*means clustering algorithm. The number of probes per cluster is denoted in the title of each plot.

**SUPPLEMENTARY FIGURE 5 : Age-positions are not consistent in permuted datasets**

Age

Age

Original dataset

Permuted dataset


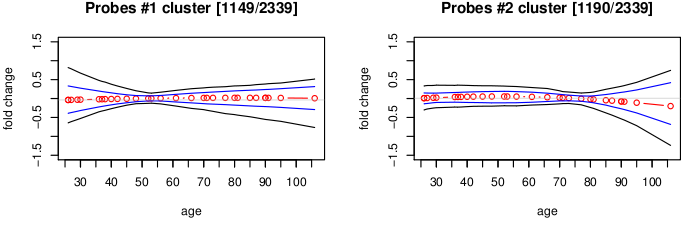

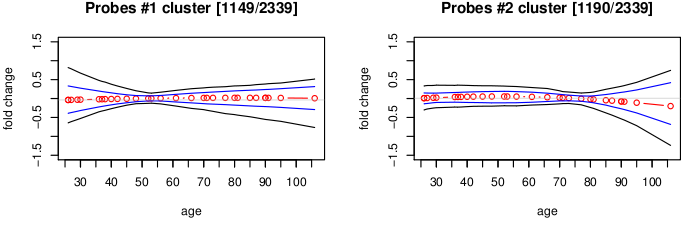

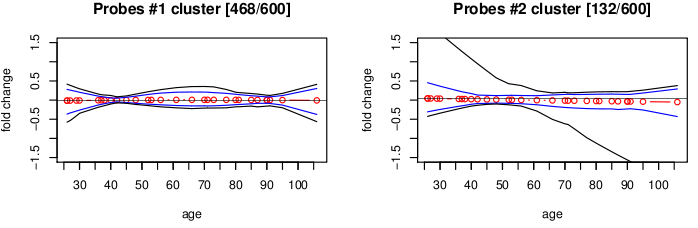

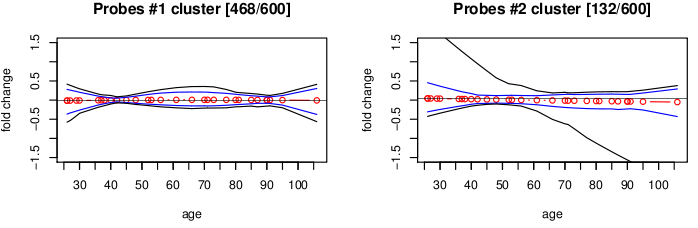


**Cluster #1 [1149 / 2339]**

Age

**Cluster #2 [1190 / 2339]**

Age

**Cluster #1 [468 / 600]**

**Cluster #2 [132 / 600]**

Fold change (log2)

**A**

Fold change (log2)

Fold change (log2)

Fold change (log2)


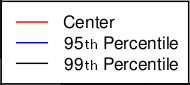


Age

Age


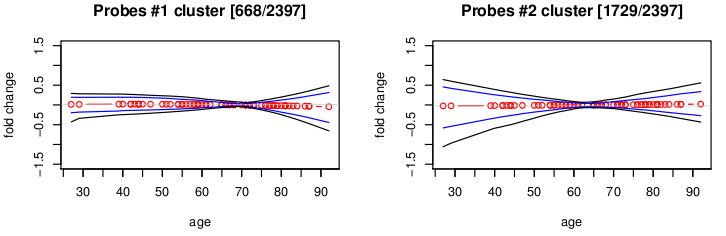

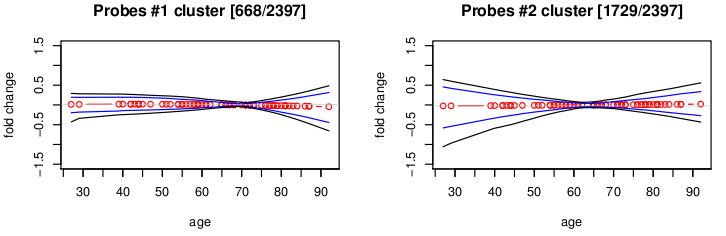


Age

Age


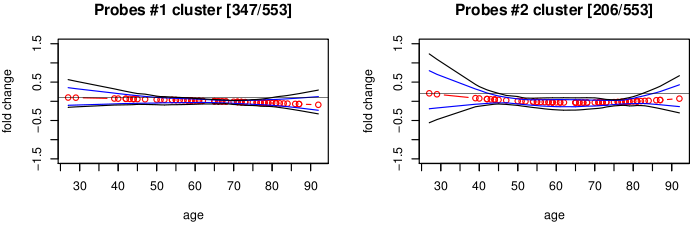

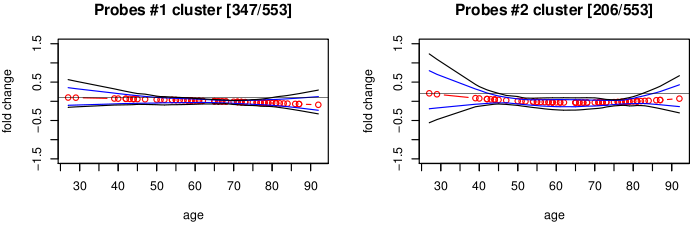


**Cluster #1 [668 / 2397]**

**Cluster #2 [1729 / 2397]**

**Cluster #2 [206 / 553]**

**Cluster #1 [347 / 553]**

Fold change (log2)

Age

Age

Original dataset

Permuted dataset

**B**


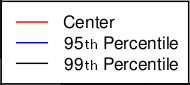


In order to determine the significance of the age-associated clusters, absolute correlation *k*-means clustering was compared between original and permuted datasets. Plots show absolute correlation clusters of the significant probes in brain cortex (A) or kidney cortex (B) datasets. Plots in the upper rows were generated from the original datasets and in lower rows from permuted datasets. The red circles interconnected with red lines represent the cluster centroids. The 95th percentile in blue depicts the dominant trends in the cluster while the 99th percentile, in black, denotes the within cluster variation. The number of probes per cluster, as opposed to the total number of probes is present in the title of each plot. Unlike the trends in the original dataset, the clusters obtained from the permuted datasets often do not show an age-association trend. Importantly, the age-positions identified in the original dataset are not reproduced in the permuted dataset. This denotes the consistency of the age associated clusters and proves that the occurrence of the age-positions in expression profiles is not an artefact of the clustering method.

**SUPPLEMENTARY FIGURE 6: Absolute correlation *k-*means clustering applied on the significant probes in *Vastus lateralis***

2^nd^ Age-position


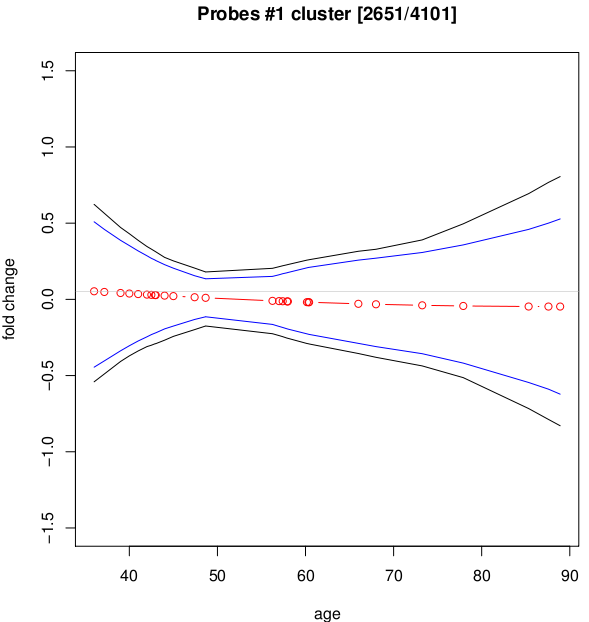

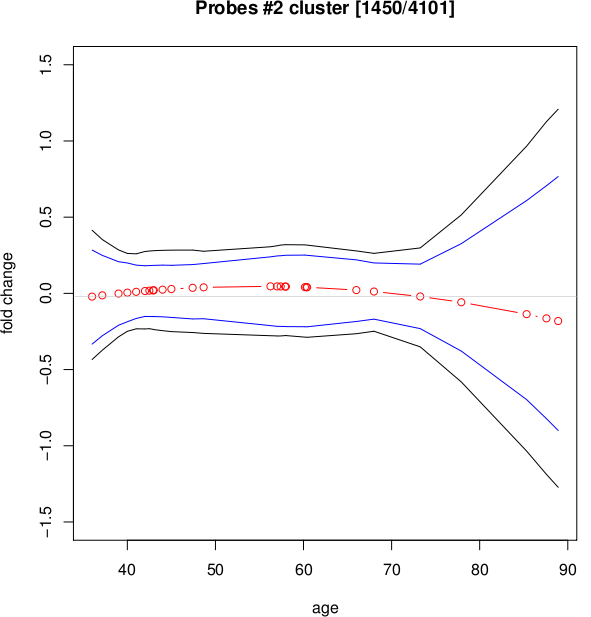


Fold change (log2)

Age

Age

**Cluster #1 [2716 / 4101]**

**Cluster #2 [1385 / 4101]**


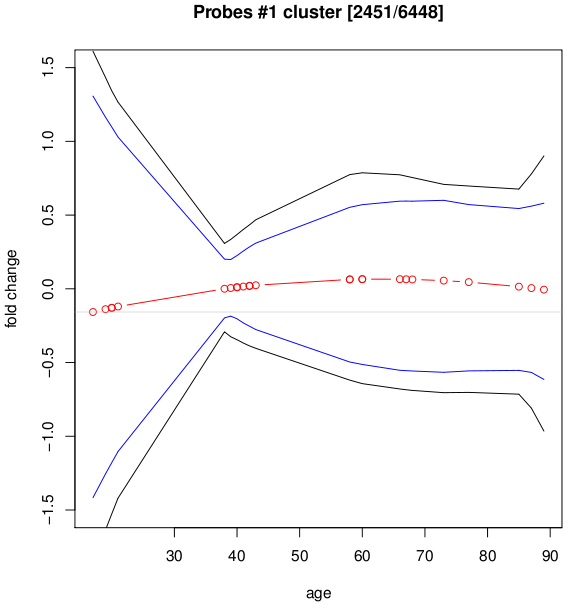

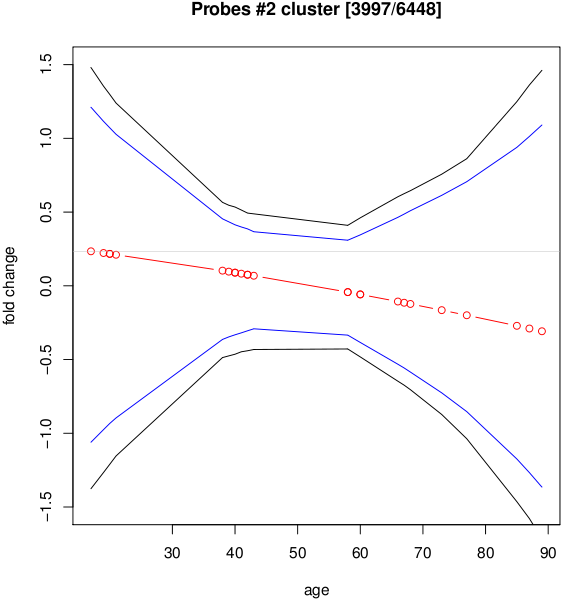


Fold change (log2)

Fold change (log2)

Fold change (log2)

**Cluster #1 [2451 / 6448]**

**Cluster #2 [3997 / 6448]**

*Vastus lateralis* B

*Vastus lateralis* A

1^st^ Age-position


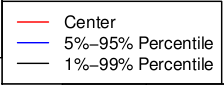

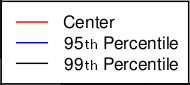


Age

Age

In order to validate absolute correlation *k*-means clustering and the occurrence of age-positions, two datasets from *Vastus lateralis* were compared: *Vastus lateralis* A (age-range 35-89, N=29) and *Vastus lateralis* B (age-range 17-89, N=25). Plots show the results of *k-*means clustering using absolute correlation as metric, applied on the significant probes of the *Vastus lateralis* dataset. The hollow red circles interconnected by the red line denote the cluster centroids. In blue, the 95th percentile shows the major trends within the cluster, while the 99th percentile in black represents the variations. The number of probes per cluster is denoted in the title of each plot. The cluster centroids depict the distribution of the up and down regulated probes within the cluster. A descending trend thus indicates a higher number of down regulated probes present in the cluster as opposed to the up regulated ones. Note that the earlier age points in *Vastus lateralis* B cause an earlier occurrence of the age-positions. This is reflected by a shift on the X axis of the age-position.

**SUPPLEMENTARY TABLES**

**SUPPLEMENTARY TABLE 1: A summary of the platform details of the datasets that were used in our study.**

| **Tissue** | **Nr of samples** | **Age range** | **Microarray platform** | **Nr of annotated probes (%)** | **Unique Entrez ID (%)** |
| --- | --- | --- | --- | --- | --- |
| ***Vastus lateralis* muscles** | 29 | 35-89 | Illumina HumanWG-6 v3.0 | \| 36375 \| \| --- \|   (74.5) | 24151  (36.9) |
| **Post-mortem brain cortex** | 30 | 26-106 | Affymetrix U95 V2 | \| 11750 \| \| --- \|   (93.5) | 8818  (65.6) |
| **Kidney cortex** | 70* | 27-92 | Affymetrix U133A | \| 20357 \| \| --- \|   (91.4) | 12718  (52.2) |
| **Kidney medulla** | 60^#^ | 29-92 | Affymetrix U133A | \| 20357 \| \| --- \|   (91.4) | 12718  (52.2%) |

The table summarizes details of the datasets used in this study: the number of samples, the age range, the microarray platform, the number of probes that were spotted on each platform, as well as the number of unique Entrez IDs. The percentages they represent from the total number of probes are indicated in parenthesis. *A duplicated sample from the original source is excluded. ^#^ A sample where gender could not be determined is excluded.

**SUPPLEMENTARY TABLE 2: Age distribution per decade in four datasets that were used for trend analysis.**

|  | 2^nd^  (11 – 20) | 3^rd^  (21 – 30) | 4^th^  (31 – 40) | 5^th^  (41 – 50) | 6^th^  (51 – 60) | 7^th^  (61 – 70) | 8^th^  (71 – 80) | 9^th^  (81 – 90) | 10^th^ and up (>90) | **Total** |
| --- | --- | --- | --- | --- | --- | --- | --- | --- | --- | --- |
| *VL* muscle (35-89 years) | 0 | 0 | 5 | 9 | 8 | 2 | 2 | 3 | 0 | 29 |
| Brain | 0 | 5 | 4 | 3 | 3 | 3 | 4 | 5 | 3 | 30 |
| Kidney cortex | 0 | 2 | 2 | 9 | 15 | 13 | 20 | 10 | 1 | 72 |
| Kidney cortex (half dataset) | 0 | 1 | 1 | 5 | 7 | 6 | 10 | 5 | 1 | 36 |
| Kidney medulla | 0 | 1 | 2 | 7 | 13 | 9 | 18 | 10 | 1 | 61 |
| *VL* muscles (17-89 years) | 4 | 1 | 4 | 4 | 4 | 3 | 2 | 3 | 0 | 25 |

The distribution of samples is shown per decade.

**SUPPLEMENTARY TABLE 3. Overlap of age-associated Entrez ID between tissues**

| **A** |  | ***VL* muscles** | **Brain** | **Kidney cortex** | **Kidney medulla** | | **B** | **Datasets** | **Entrez ID**  **(%)** |
| --- | --- | --- | --- | --- | --- | --- | --- | --- | --- |
|  | ***VL* muscles** | 100% (3235) | 13.2% (296) | 13.65%  (303) | 12.79%  (168) | |  |  |  |
|  | **Brain** | 9.1%  (296) | 100% (2238) | 16.0%  (356) | 14.31%  (188) | |  | *VL* *vs*. Brain | 296 (9.14%) |
|  | **Kidney cortex** | 9.4%  (303) | 15.9% (356) | 100%  (2219) | 19.42%  (255) | |  |  |  |
|  | **Kidney medulla** | 5.2%  (168) | 8.4%  (188) | 11.5%  (255) | 100%  (1313) | |  | *VL* *vs*. B *vs*. kidney cortex | 57 (1.76%) |
|  |  |  | |  |  |  |  |  |  |
|  |  |  | |  |  |  |  | *VL* *vs*. B *vs*. kidney cortex vs. kidney medulla | 4 (0.12%) |

Tables show the overlap of the significant (*p*<0.05) age-associated Entrez ID between every two-tissue combination (A), and between 2, 3 or 4 tissues (B). The number and percentages representing the gene overlap are indicated. Results are not corrected for multiple testing, thus overlaps may contain false positives.

**SUPPLEMENTARY TABLES 4-7.** Gene lists of Entrez ID and their gene symbols, for genes that are associated with the early (Tables 1 and 2) or late (Tables 3 and 4) age position in Brain cortex (Tables 1 and 3) or *VL* muscles (Tables 2 and 4), as well as lists of the overlapping genes per age-position.

**SUPPLEMENTARY TABLES 8-11.** Hierarchical clustering per tissue and per age-position of the significant GO terms.

REFERENCES

1. Krishna K, Narasimha Murty M (1999) Genetic K-means algorithm. IEEE transactions on systems, man, and cybernetics Part B, Cybernetics : a publication of the IEEE Systems, Man, and Cybernetics Society 29: 433-439.

2. Lu Y, Lu S, Fotouhi F, Deng Y, Brown SJ (2004) Incremental genetic K-means algorithm and its application in gene expression data analysis. BMC bioinformatics 5: 172.
